# Anodal Transcranial Direct Current Stimulation (TDCS) at Fp3–Fp2 Reduces the Face Inversion Effect: Evidence Against an Expectancy Account

**DOI:** 10.3390/brainsci16091000

**Published:** 2026-09-21

**Authors:** Ciro Civile

**Affiliations:** Department of Psychology, Faculty of Health & Life Sciences, University of Exeter, Washington Singer Building, Perry Road, Exeter EX4 4QG, UK; c.civile@exeter.ac.uk

**Keywords:** face inversion effect, perceptual learning, transcranial direct current stimulation, sham control, face recognition

## Abstract

Background: Anodal transcranial direct current stimulation (tDCS) delivered through the Fp3–Fp2 montage reduces the face inversion effect (FIE), a result now used to argue that perceptual expertise underlies face recognition. Because beliefs about brain stimulation can change behaviour in the absence of any current, the literature rests on a premise that has never been examined: its sham condition provides a neutral baseline. The objective of this study was to test that premise, by establishing whether taking part in a tDCS study, as against no such study, changes performance in this paradigm. Methods: Participants (*n* = 96, 32 per group) completed an old/new recognition task with upright and inverted faces. The anodal and sham groups were run under a double-blind procedure, so that participants in both knew they might be receiving active stimulation; a third, no-tDCS group completed the identical task on the same timetable, with no electrodes applied and no mention of stimulation. Results: Anodal tDCS halved the FIE relative to both control groups (*M* = 0.23 vs. 0.55 and 0.56), where *F*(2, 93) = 9.99, *p* < 0.001, and ηp2 = 0.18, through impaired recognition of upright faces, where *p* < 0.001; sensitivity to inverted faces was unaffected, where *p* = 0.983. The sham and no-tDCS groups were equivalent on every measure taken, and every Bayes factor for that comparison favoured the null (BF = 0.18–0.27). Conclusions: Within the paradigm and procedure employed here, the beliefs that accompany taking part in a tDCS study did not change performance. The reduction in the FIE by anodal tDCS at Fp3–Fp2 is therefore unlikely to be an expectancy effect, and the sham control used throughout this literature study can be treated as a valid baseline.

## 1. Background

Turning a face upside down produces a disproportionate cost to recognition, one far larger than the cost incurred by inverting most other classes of visual stimuli [1,2,3], and inversion appears to change the way a face is perceived rather than merely to make it harder to see [4]. Two broad accounts of this face inversion effect (FIE) have competed for several decades, and the question remains open. Specificity accounts hold that the mechanisms involved are recruited selectively for faces and play little or no role in the processing of other stimuli [5]. In their support, objects of expertise have repeatedly failed to show the signatures of face-like processing when the comparison is made under matched conditions [6], and meta-analysis suggests that the holistic effects obtained for non-face categories are smaller and less consistent than those obtained for faces [7]. Expertise accounts hold instead that they are engaged by any class of stimuli sharing a common configuration, provided the observer has sufficient experience with that class: dog experts show an inversion effect for dog images [8], and an inversion effect emerges for artificial objects, the Greebles, only after training [9]. Critically for present purposes, McLaren [10] showed that brief pre-exposure to checkerboard exemplars from a prototype-defined category was sufficient to generate an inversion effect for novel exemplars of that category, a result later obtained in the old/new recognition paradigm conventionally used to demonstrate the FIE for faces [11].

The theoretical basis for these findings is the McLaren–Kaye–Mackintosh (MKM) model of stimulus representation [12,13,14]. On this account, the elements representing features common to members of a category are more frequently encountered and more reliably predicted than those unique to a given exemplar; they therefore form strong associations rapidly and their salience declines. Because it is the unique elements that support discrimination between exemplars, the net consequence of experience with a category is perceptual learning. Inverting a stimulus removes it from the category for which this redistribution of salience has taken place, so the advantage is lost and an inversion effect results. The model thus identifies the FIE for faces as, at least in part, an instance of that general phenomenon rather than the product of a dedicated face-processing module.

Over the past decade this account has been given a causal test using transcranial direct current stimulation (tDCS). Prefrontal tDCS had already been shown to facilitate probabilistic category learning [15] and to accelerate the learning of difficult perceptual discriminations [16]. Adopting the montage first used by Ambrus et al. [17] to impair categorisation in a prototype distortion task, Civile et al. [18] found that anodal stimulation delivered over the left dorsolateral prefrontal cortex (DLPFC) at Fp3 eliminated the inversion effect for familiar checkerboards. Civile et al. [19] extended the procedure to faces in three double-blind experiments, in two of which the robust FIE obtained under sham was significantly reduced under anodal stimulation at Fp3–Fp2, the reduction being carried by impaired recognition of upright faces with no difference between the groups for inverted faces. Two features of this result matter for what follows. The first is direction: anodal tDCS delivered through the Fp3–Fp2 montage reduces the FIE, and it does so by making performance worse rather than better. The second is selectivity: within a session containing both orientations, the effect is confined to upright stimuli, with overall performance being unaffected [20,21], whereas where every stimulus is an upright face overall performance does fall [22].

The anatomical specificity of the Fp3–Fp2 montage has been established through a series of active control experiments, each holding the behavioural task, the stimulation parameters and the double-blind procedure constant while moving the anode, the return electrode, or both: the right inferior frontal gyrus (rIFG) with the return above the left eyebrow at Fp1 [19], the occipital site PO8 with the return at Fp2 [22], and Fp3–Cz, with the anode unchanged and only the return electrode relocated to the vertex [23]. The generalisation supported by this series is a precise one: every active control group tested has behaved like sham, and the reduction in the FIE has been obtained with the Fp3–Fp2 montage and with no other montage yet set against it.

What the stimulation does to perceptual learning has been given a specific interpretation within the MKM framework: anodal tDCS at Fp3–Fp2 is taken to disable the error-based modulation of salience on which perceptual learning depends, so that experience with a category comes to promote generalisation between its members rather than discrimination among them. Civile et al. [24] implemented this account in simulations and confirmed the counterintuitive prediction that follows from it: the same stimulation that reduces the FIE when upright faces are presented among other normal faces increases it when they are presented among Thatcherised faces, from which harmful generalisation would otherwise flow. The effect is therefore not a blunt impairment of face recognition but a specific interference with the salience modulation on which perceptual learning depends. The protocol has since been used as an instrument rather than as an object of study, to argue that the other-race effect [20] and the own-age bias [21] each depend on perceptual expertise, in both cases through a sham-relative reduction in the FIE for the more familiar face category.

All these rest on the assumption that what the Fp3–Fp2 montage delivers is neuromodulation rather than expectation. That assumption deserves scrutiny, because a substantial literature study now shows that beliefs about brain stimulation can change behaviour in the absence of any current. Braga et al. [25], reviewing 30 studies, argue that sham protocols control participants’ beliefs about which condition they are in without controlling their prior beliefs about what stimulation does, and they document repeated failures of blinding integrity at conventional intensities, a problem since confirmed in independent samples [26]. The most striking single demonstration is that of Ray et al. [27]: in a balanced-placebo design, simply telling participants they had received real tDCS substantially reduced how much they subsequently ate, while the stimulation itself had no detectable effect at all. Such expectations are readily installed, a single brief written prime being enough to open a wide gap between participants primed to expect benefit and those primed to expect none [28]. They can also interact with genuine stimulation in ways that are far from additive. Allaert et al. [29], in a large double-blind study using an F3–Fp2 montage, found that active stimulation under neutral expectations and positive expectations under sham each reduced the anti-saccade accuracy cost, but that combining the two conferred no advantage at all. The picture is not uniform. Rabipour et al. [30] shifted expectations successfully but found no consequence for performance, and Hanson et al. [31] reported neither placebo nor nocebo effects on cycling performance. Nonetheless, the adequacy of sham control is now explicitly identified by the field as an unresolved methodological problem [32].

What can be drawn from the existing record is more limited than it first appears. Two observations sit against an expectancy account of the Fp3–Fp2 effect on perceptual learning and the FIE. The first is selectivity: an expectancy effect might plausibly express itself as a change in overall performance, effort or engagement; yet in designs containing both orientations this protocol acts only on upright stimuli, sparing inverted stimuli within the same session and the same participants. The second is the behaviour of the active controls: participants in the rIFG, PO8 and Fp3–Cz groups were run under the same double-blind procedure, with the same apparatus and the same instructions as those in the Fp3–Fp2 groups, and performed at sham levels [19,22,23]. Neither, however, constitutes a test. Expectation has never been measured in any study using this protocol, and in every one of them the comparison against which the anodal effect was measured was a sham group, that is, a group of participants who knew they were taking part in a brain stimulation experiment and who, under double-blind allocation, had every reason to believe they might be receiving active stimulation. Whether that belief itself changes performance in this paradigm has never been asked.

The present study asks it. Using the same double-blind, between-subjects design and the same old/new recognition task with upright and inverted faces employed in the original study by Civile et al. [19] (Experiments 1 and 2), we compared three groups. The anodal group received the standard Fp3–Fp2 protocol. The sham group received the standard sham procedure with the same montage in place. The third group, the no-tDCS group, completed the identical behavioural task on exactly the same timetable, but with no electrodes applied and with no mention of brain stimulation at any point. It should be stated at the outset what this design can and cannot establish. Expectations were not measured directly. The comparison tests whether taking part in a tDCS study, as against taking part in no such study, affects performance; it does not test whether experimentally induced expectations modulate the effects of tDCS, and the no-tDCS group isolates the beliefs that accompany a stimulation context rather than placebo and nocebo processes in general. Two competing predictions follow, and they turn on the comparison between the sham and no-tDCS groups rather than on the anodal group alone. The expectations generated by taking part in a brain stimulation study fall on the sham group as heavily as on the anodal group, since under double-blind allocation neither knows which it is. If those expectations contribute to performance in this paradigm, the sham group should depart from the no-tDCS baseline, and the sham condition against which a decade of anodal effects has been measured would not be the neutral comparison it has been assumed to be. If, on the other hand, the Fp3–Fp2 effect reflects genuine neuromodulation of the mechanisms supporting perceptual learning, then the sham and no-tDCS groups should be indistinguishable at both orientations, and only the anodal group should show a reduced FIE, a reduction carried by impaired recognition of upright faces, with inverted faces unaffected.

## 2. Methods

### 2.1. Participants

Ninety-six naïve participants (55 women; *M*_age_ = 22.34 years, *SD* = 2.81, range: 18–31) took part in the study, with 32 randomly assigned to each of the three groups. The groups did not differ in age, where *F*(2, 93) = 0.02 and *p* = 0.985 (anodal, *M* = 22.41, *SD* = 2.86, 18 women; sham, *M* = 22.34, *SD* = 2.72, 18 women; no-tDCS, *M* = 22.28, *SD* = 2.94, 19 women). All participants were students from the University of Exeter and were given course credits or cash for their participation. Only right-handed individuals with no history of neurological or psychiatric conditions, no metal implants in the scalp and no pacemaker, who were not pregnant, had no skin conditions and were not taking medication took part in the study. The restriction to right-handed participants reflects the left-lateralised placement of the anode: handedness is associated with differences in the functional lateralisation of the frontal cortex [33,34], so admitting left-handed participants would introduce variance in the relation between a fixed electrode position and the underlying functional anatomy. Informed consent was obtained from all participants, and the research was approved by the Psychology Research Ethics Committee at the University of Exeter.

The sample size was determined by an a priori power analysis conducted in G*Power 3.1 [35] for the group × orientation interaction of the 2 × 3 mixed analysis of variance (ANOVA: repeated measures, within–between interaction; α = 0.05, power = 0.80, three groups, two measurements, correlation among repeated measures set to zero and nonsphericity correction ε = 1). With a conservative medium effect size assumed, *f* = 0.25, smaller than the interaction effects obtained in the studies that established this protocol, which corresponded to *f* = 0.35 and *f* = 0.42; the analysis indicated that a minimum of 81 participants would be required. Ninety-six were recruited, 32 per group, which exceeded that requirement and afforded a power of 0.87 to detect an interaction of medium size and in excess of 0.99 to detect one of the sizes previously reported for this montage. The sample size is also consistent with that used in previous studies employing the same behavioural paradigm and tDCS montage [19,36].

The design additionally took into account the recommendations of Brysbaert and Stevens [37] regarding statistical power in repeated measures designs. On the basis of sampling simulations from two megastudies, those authors concluded that approximately 1600 observations, across participants and trials, in the smallest analysis cell are sufficient to detect smaller-than-medium effects with power exceeding 0.80. In the present study the smallest analysis cell, one group at one orientation, comprised 64 observations from each of the 32 participants, or 2048 observations; across the full sample each orientation contributed 64 observations from each of the 96 participants, or 6144 observations.

### 2.2. Materials

The study used a set of 128 male and 128 female face images standardised to greyscale on a black background employed by Civile et al. [19] and in previous studies using this old/new recognition paradigm. The stimuli measured 5.63 cm × 7.84 cm and were presented at a resolution of 1280 × 960 pixels. The experiment was run using Superlab 4.0.7b on an iMac computer, with participants seated approximately 70 cm from the screen on which the images were presented.

### 2.3. Behavioural Procedure

The behavioural procedure was identical to that used in Experiments 1 and 2 by Civile et al. [19]. The old/new recognition task consisted of two parts, a study phase and an old/new recognition phase. In the study phase each participant was shown 64 upright and 64 inverted male and female face stimuli, 128 images in total, presented one at a time in random order with no response required. Each trial began with a fixation cross at the centre of the screen for 1 s, followed by a face stimulus presented for 3 s before the programme moved on to the next trial.

After all 128 study stimuli had been presented, the programme displayed a further set of instructions explaining the recognition task. In the recognition phase, 128 novel face stimuli (half upright, half inverted) were added to the 128 stimuli seen during the study phase, and all 256 images were presented one at a time in random order. Participants pressed the ‘.’ key if they recognised the stimulus as having been shown in the study phase and the ‘x’ key if they did not, with the two keys counterbalanced across participants. Each face was presented for 3 s, within which the response had to be made. For a given participant, each face stimulus appeared in only one orientation, upright or inverted, throughout the experiment, and the stimulus sets were rotated across participants in a counterbalanced manner, so that each face seen upright by a participant in one group was seen upright equally often by participants in the other two groups.

### 2.4. TDCS Apparatus

**Figure 1:**
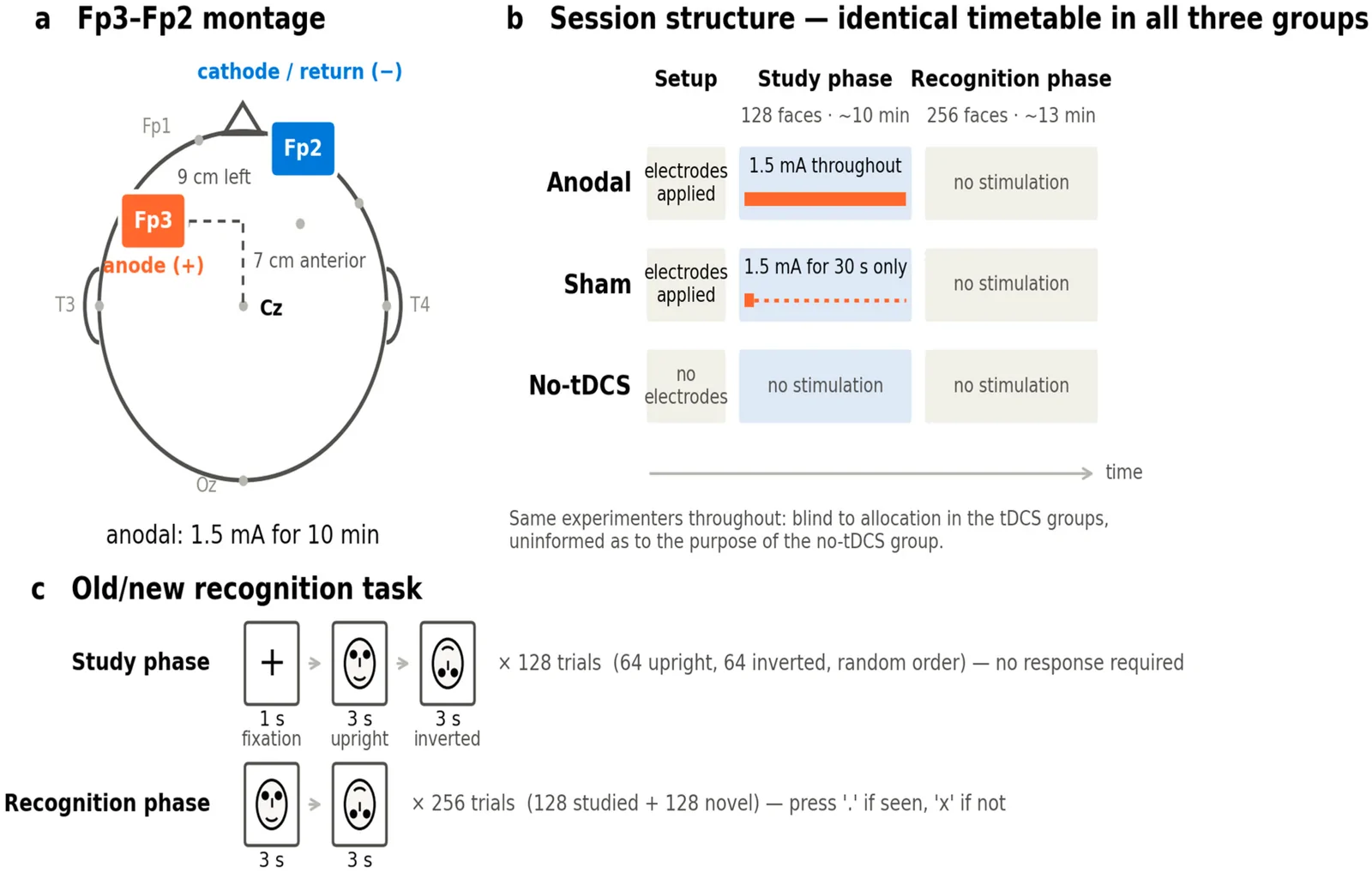
tDCS montage, session structure and behavioural task. (**a**) The Fp3–Fp2 montage. The anode was placed over Fp3, located 7 cm anterior and 9 cm to the left of Cz, and the cathode/return electrode over the contralateral supraorbital area at Fp2. Grey markers show 10–20 sites for orientation. Electrodes were 7 cm × 5 cm saline-soaked sponges (35 cm^2^). (**b**) Session structure. All three groups followed the same timetable; the anodal and sham groups wore the montage and were run under the double-blind protocol, whereas the no-tDCS group had no electrodes applied. All three groups were run by the same experimenters, who were blind to allocation in the anodal and sham conditions and uninformed as to the purpose of the third. (**c**) The old/new recognition task. Stimulation covered the study phase only.

For the anodal and sham groups, stimulation was delivered by a battery-driven constant current stimulator (neuroConn DC-Stimulator Plus) using a pair of surface sponge electrodes (7 cm × 5 cm, i.e., 35 cm^2^) soaked in saline solution and applied to the scalp at the target areas of stimulation. We adopted the Fp3–Fp2 montage used by Civile et al. [19] (Experiments 1 and 2): a bilateral bipolar non-balanced arrangement with one electrode (the anode) placed over the target stimulation area at Fp3 and the other (the cathode/return) on the forehead over the contralateral supraorbital area at Fp2, above the right eyebrow. Fp3 was located for each participant by first identifying Cz, half the distance between the inion and the nasion, and then measuring 7 cm anterior relative to Cz and 9 cm to the left [17,18]. The montage, the session structure and the behavioural task are illustrated in Figure 1.

Because the purpose of this study was to evaluate the sham condition of an existing protocol, any departure from that protocol’s parameters would confound the comparison, and they were therefore fixed to reproduce the study by Civile et al. [18,19] exactly. Each nonetheless has a rationale of its own. Constant direct current was used rather than an alternating or pulsed waveform because the mechanism at issue is a sustained, polarity-dependent shift in cortical excitability brought about by subthreshold modulation of the resting membrane potential [38], rather than entrainment of an endogenous oscillation, which is what alternating-current protocols are designed to achieve. The intensity of 1.5 mA and the duration of 10 min, with 5 s fade-in and 5 s fade-out, follow the originating studies; the ramps serve the additional purpose of reducing the cutaneous sensation at onset and offset, which is the principal cue by which participants can distinguish active from sham stimulation [39]. The rectangular sponge electrodes of 7 cm × 5 cm (35 cm^2^) yield a current density of 0.043 mA/cm^2^ at the scalp and a total charge density of 25.7 mC/cm^2^ across the stimulation period, both within the limits set out in current safety guidance [40] and within the range routinely employed in cognitive tDCS research [41]. Sponge electrodes of this size trade spatial focality for a lower current density and better tolerability. Focality was not a design objective here, because the anatomical specificity of this montage had been established behaviourally, through the active control experiments described above, rather than inferred from electrode geometry.

The anodal and sham conditions were run using a double-blind procedure reliant on the neuroConn study mode, in which the experimenter inputs numerical codes provided by another experimenter otherwise unconnected with running the experiment; these codes switch the stimulation mode between “normal” (i.e., anodal) and “sham” without revealing which has been selected. In the anodal condition, a direct current of 1.5 mA was delivered for 10 min (5 s fade-in and 5 s fade-out), starting as soon as the participant began the behavioural task and continuing throughout the study phase only. In the sham condition, the identical stimulation mode was displayed on the stimulator and participants experienced the same 5 s fade-in and 5 s fade-out, but the 1.5 mA current was delivered for just 30 s, following which a small current pulse (3 ms peak) was delivered every 550 ms (0.1 mA over 15 ms) for the remainder of the 10 min in order to check impedance levels.

In both conditions the stimulation began as soon as the participant started the study phase and was terminated at the end of it. The study phase comprised on-screen instructions followed by 128 trials, each consisting of a 1 s fixation cross and a 3 s face presentation; the trials themselves occupied approximately 8.5 min, and the phase as a whole spanned the 10 min of stimulation. No stimulation was delivered during the recognition phase that followed, although the tDCS electrodes remained on the participant throughout [19]. It is important to note that the after-effects of tDCS persist for up to 120 min post-stimulation [38,42], so that the stimulation delivered during the study phase remains effective across the recognition phase.

Immediately after the session, and following the standard post-stimulation safety screening, participants in the anodal and sham groups completed a questionnaire on the sensations they had experienced during stimulation. No significant differences emerged between the two groups on any item, indicating that the sham procedure could not be distinguished from active stimulation on the basis of what participants felt. This accords with earlier work establishing that the fade-in, short-stimulation, and fade-out sham adopted here is not reliably distinguishable from active stimulation by participants, whether naïve to the technique or experienced with it [39,43].

Participants in the no-tDCS group had no electrodes applied and received no information about brain stimulation at any point. Apart from the absence of the montage, their sessions followed exactly the same timetable as those of the anodal and sham groups, so that the intervals between arrival, the study phase and the recognition phase were matched across the three groups. All three groups were run by the same experimenters. For the anodal and sham groups those experimenters were blind to allocation, as described above; for the no-tDCS group they were not informed of the purpose the condition served, and so were in no position to convey any expectation about stimulation or its effects to the participants in it. This group removes the experimental context of brain stimulation and the beliefs that accompany participation in it: the electrodes, the stimulator, the information sheet describing tDCS, and the knowledge that current may be flowing. Its scope is correspondingly specific: it affords a comparison between the presence and the absence of a stimulation context rather than between the presence and absence of expectation as such, since verbal suggestion, conditioning and the framing of the intervention were held constant across the three groups rather than manipulated.

### 2.5. Data Analysis

For each participant, hits and false alarms were tabulated separately for upright and inverted faces from the 256 trials of the recognition phase, which comprised 64 studied and 64 novel stimuli at each orientation. No participant was excluded and no data were missing: all 96 participants contributed complete data at both orientations. No hit rate or false-alarm rate fell at 0 or 1, so no correction for extreme values was required [44].

### 2.6. Evaluation Metrics

Recognition performance was indexed by *d*′, the signal detection theory measure of sensitivity, computed for each participant at each orientation as *z*(hit rate) − *z*(false-alarm rate) [44]. It is also the measure used throughout the previous studies that employ this old/new recognition paradigm and this stimulation protocol [11,18,19], so that the present results are directly comparable with theirs. *d*′ was preferred to percentage correct because it separates sensitivity from response bias, a distinction that carries particular weight here: the hypothesis under test concerns participants’ beliefs, and such beliefs might plausibly act on a participant’s willingness to declare a face “old” rather than on the ability to discriminate studied from novel faces. The primary dependent measure was the face inversion effect, defined within participants as upright *d*′ minus inverted *d*′. Three further measures derived from *d*′ were analysed: upright *d*′, inverted *d*′ and overall *d*′, the mean of the two orientations. Larger values of *d*′ denote greater sensitivity, and larger values of the inversion effect a greater upright advantage.

### 2.7. Statistical Analysis

The *d*′ scores were submitted to a 2 (face orientation) × 3 (group) mixed ANOVA, with orientation as a within-subjects factor and group as a between-subjects factor (*n* = 32 per group). Model assumptions were checked before interpretation and are reported at the beginning of the Section 3. Two families of the follow-up comparisons were corrected for multiple testing. The three within-group tests of the FIE, one per group, were Bonferroni-corrected for three comparisons. All pairwise between-group comparisons were Tukey-corrected within each dependent measure, namely the FIE, upright *d*′, inverted *d*′ and overall *d*′, with three comparisons in each family. Omnibus *F* tests are reported uncorrected, as is the single planned contrast comparing the anodal group with the two control groups combined. Effect sizes are reported as partial η^2^ for ANOVA terms, *d*_z_ for within-subjects contrasts and Hedges’ *g* for between-group contrasts, with 95% confidence intervals for the mean inversion effects. Bayes factors were computed both with an informed half-normal prior and with the default Jeffreys–Zellner–Siow prior, and a sensitivity analysis across prior scales was conducted; that procedure is set out where the Bayes factors are first reported.

## 3. Results

Recognition sensitivity was indexed by *d*′, computed for each participant as *z*(hit rate) − *z*(false-alarm rate) separately for upright and inverted faces. No hit rate or false-alarm rate fell at zero or one, so no correction was required. Cell means and standard deviations are presented in Table 1. The *d*′ scores were submitted to a two (face orientations: upright, inverted) × three (groups: anodal, sham, no-tDCS) mixed analysis of variance (ANOVA), with orientation as a within-subjects factor and group as a between-subjects factor (*n* = 32 per group). Preliminary checks supported the model: variances were homogeneous at both orientations (Levene’s tests, *p*-values ≥ 0.237), and the covariance matrices did not differ across groups, where Box’s *M*, χ^2^(6) = 2.59, and *p* = 0.859. Sphericity was not at issue because the within-subjects factor comprised only two levels.

The inversion effect is the within-participant difference between upright and inverted *d*′, where *d*′ = *z*(hit rate) − *z*(false-alarm rate) with *n* = 32 in each group.

Bayes factors (BFs) were computed following the convention adopted in previous works with this protocol [45], using a half-normal prior whose standard deviation was set from the relevant earlier finding. For comparisons of the anodal group with either control group, the prior was scaled to the mean sham-minus-anodal difference in the FIE reported by Civile et al. [19] (Experiment 1), at 0.26. For comparisons between the two control groups it was scaled to the size of the anodal effect obtained here, at 0.32, on the reasoning that if belief alone was sufficient to produce the effect it should do so at the magnitude produced by the current. Values above three are taken as evidence for the alternative hypothesis and values below 1/3 as evidence for the null [46]. Default Jeffreys–Zellner–Siow Bayes factors [47] are reported alongside, expressed as BF_01_ where they favour the null. All Bayes factors for the between-group comparisons are given in Table 2.

The ANOVA yielded a large main effect of face orientation, where *F*(1, 93) = 171.98, *p* < 0.001, and η_p_^2^ = 0.65, with higher sensitivity for upright (*M* = 0.66, *SD* = 0.29) than for inverted faces (*M* = 0.21, *SD* = 0.23). The main effect of group was also significant, where *F*(2, 93) = 9.22, *p* < 0.001, and η_p_^2^ = 0.17, as was the group × orientation interaction, where *F*(2, 93) = 9.99, *p* < 0.001, η_p_^2^ = 0.18, and BF_10_ = 118 by the Bayesian information criterion (BIC) approximation [48]. Because the interaction qualified both main effects, it was decomposed in two complementary ways.

First, the FIE (upright *d*′ − inverted *d*′) was examined within each group. A reliable FIE was obtained in all three, but it was substantially smaller following anodal stimulation (*M* = 0.23, 95% confidence interval (CI) [0.11, 0.35]), where *t*(31) = 3.85, *p* = 0.002, and *d*_z_ = 0.68, than following sham (*M* = 0.55, 95% CI [0.44, 0.66]), where *t*(31) = 10.07, *p* < 0.001, and *d*_z_ = 1.78, or in the no-tDCS group (*M* = 0.56, 95% CI [0.43, 0.68]), where *t*(31) = 9.02, *p* < 0.001, and *d*_z_ = 1.59; these three *p*-values are Bonferroni-corrected. A one-way ANOVA confirmed that the magnitude of the FIE differed across groups, where *F*(2, 93) = 9.99, *p* < 0.001, and η_p_^2^ = 0.18. Tukey-corrected comparisons showed that the anodal group differed from both the sham group, with *p* = 0.001, *g* = 0.97, and BF = 659, and the no-tDCS group, with *p* < 0.001, *g* = 0.94, and BF = 401, whereas the sham and no-tDCS groups did not differ, where *p* = 0.995, *g* = 0.02, BF = 0.27, and BF_01_ = 3.91. A planned contrast comparing the anodal group with the two control groups combined confirmed the reduction, where *t*(60.06) = 4.44, *p* < 0.001, and *d* = 0.97 (mean difference: 0.32, 95% CI [0.18, 0.47]).

Second, the effect of group was examined at each orientation separately. For upright faces, sensitivity differed reliably across groups, where *F*(2, 93) = 17.87, *p* < 0.001, and η_p_^2^ = 0.28: the anodal group was less sensitive than both the sham group, with *p* < 0.001, *g* = 1.33, and BF > 1000, and the no-tDCS group, where *p* < 0.001, *g* = 1.28, and BF > 1000, which did not differ from one another, where *p* = 0.952, BF = 0.25, and BF_01_ = 3.77. For inverted faces, by contrast, the three groups were indistinguishable, where *F*(2, 93) = 0.02, *p* = 0.983, and η_p_^2^ < 0.001, with no pairwise difference approaching significance (all *p*-values > 0.98) and every Bayes factor favouring the null (BF ≤ 0.25, all BF_01_s ≥ 3.86).

Differences are the first-named group minus the second. BF is the informed Bayes factor computed with a half-normal prior [45], scaled to the effect reported by Civile et al. [19] (Experiment 1) for comparisons involving the anodal group and to the size of the anodal effect obtained here for comparisons between the two control groups. BF_10_ is the default Jeffreys–Zellner–Siow Bayes factor [47]. For both, values above three favour the alternative hypothesis and values below 1/3 the null; BF_01_ is the reciprocal. The *p*-values in this table are from uncorrected independent-samples *t* tests; the Tukey-corrected values are reported in the text.

**Figure 2:**
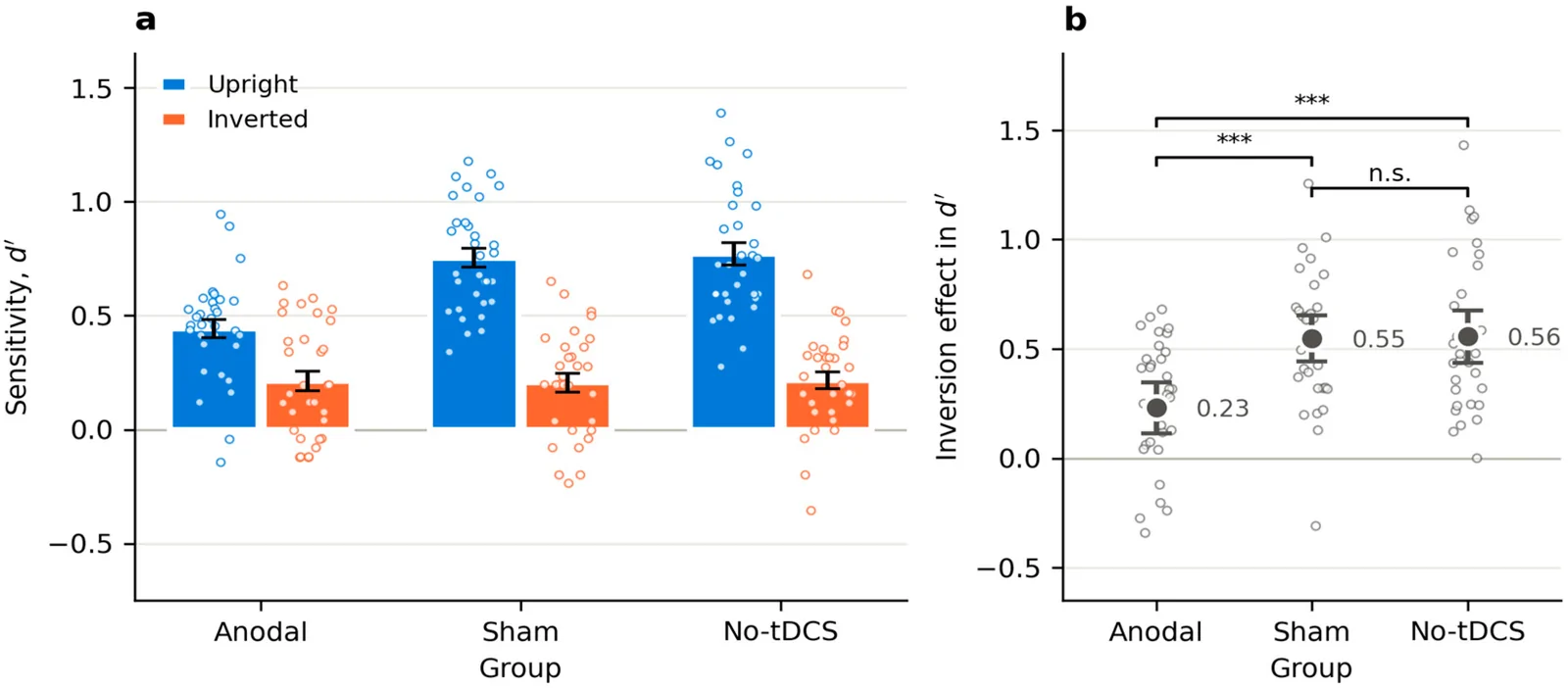
Recognition sensitivity and the face inversion effect by group. (**a**) Mean *d*′ for upright and inverted faces in each group; error bars show ±1 standard error of the mean (SEM). (**b**) The face inversion effect (upright *d*′ − inverted *d*′) in each group; error bars show 95% confidence intervals. Open circles represent individual participants (*n* = 32 per group). *** *p* < 0.001, Tukey-corrected; n.s. = not significant.

Anodal tDCS delivered through the Fp3–Fp2 montage therefore selectively reduced sensitivity to upright faces while leaving sensitivity to inverted faces unchanged, halving the FIE relative to both control groups; this pattern is displayed in Figure 2. The main effect of group reported above reflects the same selective reduction: collapsed across orientation, the anodal group showed lower overall sensitivity than the sham, where *p* = 0.002, *g* = 0.89, and BF = 167, and no-tDCS groups, where *p* < 0.001, *g* = 0.96, and BF = 515, which did not differ from each other, where *p* = 0.940, BF = 0.18, and BF_01_ = 3.73. Critically for the question motivating this study, the sham and no-tDCS groups were equivalent on every measure taken, and the Bayes factors favoured the null on all four, ranging from 0.18 to 0.27 against the 1/3 threshold, with default factors favouring the null by between 3.7 and 3.9. This conclusion is not an artefact of the prior: the Bayes factor for the FIE comparison continues to favour the null for any prior scale above 0.25, that is, whenever belief is expected to produce at least about four-fifths of the effect the current produces, and below that value it becomes inconclusive rather than reversing. No difference between the sham and no-tDCS groups was therefore detectable on any measure taken.

## 4. Discussion

The present study asked whether the reduction in the FIE produced by anodal tDCS at Fp3–Fp2 owes anything to participants’ beliefs about brain stimulation. Three groups completed the same old/new recognition task with upright and inverted faces: one received the standard anodal protocol, one the standard sham procedure under double-blind allocation, and one completed the task with no electrodes and no mention of stimulation, run by the same experimenters but with the purpose of that condition withheld from them. The anodal group produced the expected result, an FIE significantly reduced compared to that obtained in either control group, generated mainly by impaired recognition of upright faces. The two control groups were indistinguishable on every measure taken.

That second finding is the point of the study. Participants in the sham condition knew they were taking part in a brain stimulation experiment, had electrodes on their heads for the duration, felt the fade-in and fade-out of current at the start of the session, and, being blind to their allocation, had every reason to believe that they might be receiving active stimulation. Participants in the no-tDCS condition had none of this. If the context of a tDCS experiment was by itself sufficient to change performance in this paradigm, the two groups should have diverged. They did not, and the Bayesian analyses confirmed the null. With the alternative hypothesis set as belief acting on performance in the way the current did, the Bayes factors favoured the null on all four measures, the FIE, upright sensitivity, inverted sensitivity and overall sensitivity, with values between 0.18 and 0.27, below the conventional 1/3 threshold, and the default Jeffreys–Zellner–Siow factors agreed in every case, favouring the null by a factor of between 3.7 and 3.9. Nor does the conclusion depend on the prior: it holds for any prior scale above 0.25, and below that value the evidence becomes inconclusive rather than reversing. Within the paradigm and procedure employed here, the sham condition against which every anodal effect in this line of work has been measured is therefore not measurably inflated or depressed by the beliefs the procedure creates. That is the strongest statement these data support: it concerns this task, these participants and this manipulation, and not expectancy effects in tDCS generally.

This was not a foregone conclusion. The expectancy literature in non-invasive brain stimulation is substantial. Ray et al. [27] found that telling participants they had received real stimulation changed how much they subsequently ate, while the stimulation itself did nothing at all. Braga et al. [25] document repeated failures of blinding integrity at the intensities conventionally used, and argue that sham protocols control what participants believe about their allocation without controlling what they believe stimulation does. Against that background, the premise that a sham group provides a clean behavioural baseline is just that, a premise, and one that has been carried through this line of work without ever being examined.

Why, then, did expectation gain no purchase here? We suggest the answer lies in the nature of the dependent measure. An expectancy effect requires a route from belief to behaviour, and in the domains where such effects have been large that route is readily available: how much a person eats, how much pain they report, how hard they persist at a demanding task are all plausibly modulated by beliefs about efficacy, whether through motivation, effort, strategy, or the framing of a subjective judgement. The FIE offers no such route. It is not a level of performance but a difference between two levels of performance, obtained in the same session from the same participant, and it arises from a lifetime of perceptual experience with upright faces. Participants do not know that their recognition of inverted faces is being compared with their recognition of upright ones; they have no introspective access to the perceptual learning that produces the difference, and they could not strategically enlarge or suppress it if they wished to. On this view the present null is not evidence that expectancy effects are absent from tDCS research in general, nor that they are absent from this paradigm under a stronger manipulation, but evidence about which kinds of measure are vulnerable to them. It sits comfortably alongside Rabipour et al.’s [30] failure to find expectancy effects on overlearned motor performance despite having successfully shifted expectations, and alongside the contrast between that result and the large effect Ray et al. [27] obtained on a consumption decision.

The pattern of the anodal effect itself points in the same direction. Had the reduction in the FIE been driven by belief, effort or arousal, one would expect it to appear as a general shift in recognition performance. It did not. Anodal stimulation left sensitivity to inverted faces unchanged, with the three groups very similar on that measure, and acted only on upright faces, which were randomly intermixed with inverted ones throughout the test sequence. A global mechanism cannot produce an effect that respects the orientation of the stimulus under those conditions. A mechanism operating on the perceptual expertise specific to upright faces can, and that is what the MKM account predicts: anodal stimulation at Fp3–Fp2 is understood to prevent the differential loss of salience of the elements common to upright faces, so that the advantage in discriminability which experience would otherwise confer is not realised. Inverted faces, never having belonged to that category, have nothing to lose.

A different objection deserves an answer here. It might be argued that anodal stimulation impairs encoding during the study phase rather than perceptual learning, since the study phase is when the current was delivered, and that the reduced FIE simply reflects a weaker memory trace for the faces studied under stimulation. Two things speak against this. The first is again selectivity: an encoding deficit should degrade memory for inverted faces as well as upright ones, and it did not. The second is decisive. Civile et al. [49] showed across three large experiments that the effect of anodal tDCS at Fp3 can be reversed by delivering the opposite polarity, cathodal at Fp3 with the anode at Fp2, during the recognition phase. Participants who received anodal stimulation at study followed by cathodal stimulation at test showed no reduction in the FIE and no impairment for upright faces relative to a sham–sham control, whereas those who received anodal stimulation followed by sham showed the usual reduction; cathodal stimulation delivered from the outset had no effect of its own. If the anodal effect was a failure of encoding, no manipulation applied after encoding had finished could restore performance for faces that had already been studied, that it can be restored indicates that what the stimulation alters is the representation over which recognition operates, not the fidelity with which the study episode was laid down.

It is worth noting that the FIE in the anodal group, though halved, remained reliable. This replicates what Civile and Quaglia et al. [36] found using a matching task, in which the same procedure eliminated the checkerboard inversion effect but only reduced the FIE. The present data are consistent with their interpretation, on which the component removed by this procedure is the expertise-based component shared with other prototype-defined categories, while the residual reflects something specific to faces. What the present study adds is that the component the procedure removes is not itself a product of expectation: the FIE was of the same size in participants who believed they might be receiving stimulation and in participants who were never told that brain stimulation was involved in the study at all, so the expertise component that anodal tDCS abolished was present in equal measure with and without that belief.

The consequence of this result extends beyond the experiments that established the protocol. The Fp3–Fp2 procedure has since been used as an instrument: to argue that the other-race effect depends on perceptual expertise [20], and that the own-age bias does likewise [21]. In both cases the argument runs through a reduction, relative to sham, of the FIE for the more familiar face category. Had sham not been a neutral comparison, those conclusions would have inherited the problem. The present result indicates that they do not inherit it from this source.

Four directions for future work follow naturally from the present design. The first is to measure expectations rather than infer their inertness. The absence of a difference between the sham and no-tDCS groups shows that whatever expectations the sham procedure created did not translate into behaviour, but because expectations were not measured directly here it does not tell us what those expectations were, nor how much they varied between participants. Administering an expectation measure such as the Expectation Assessment Scale [28,30] alongside the task would allow the two links in that chain to be examined separately, and we would encourage it in future work with this protocol. The second is to induce an expectation rather than only to remove one. The present design contrasts participants who could believe that stimulation might be acting on them with participants for whom the question never arose, and finds no difference between them; it does not test what happens when participants are told in so many words that the stimulation will change how well they recognise faces. A balanced-placebo design of the kind used by Ray et al. [27], crossing what participants are told against what they receive, would establish whether an expectation that is explicitly installed, rather than merely available, is equally inert in this paradigm. Such a design would also reach beyond the stimulation context itself, which is what the no-tDCS group isolates, to the other routes by which a placebo or nocebo response can be produced, explicit verbal suggestion, conditioning, and the framing of an intervention, and would establish how far the present outcome extends across them. The third is to ask whether the same holds in samples with different prior beliefs. Expectancy effects depend on what participants already believe about an intervention, and a university participant pool is more likely than the general population to have encountered non-invasive brain stimulation before. Testing participants recruited outside the university, and across a wider age range, would be the most direct way of putting the sham baseline to a stronger test. The fourth is to extend the test beyond faces. The present study used face stimuli only, and we see no reason in principle to expect a different outcome for the checkerboard version of the paradigm, in which participants are if anything still less able to guess what is being measured. Running that version, together with the composite-face, other-race and other-age versions to which this protocol has also been applied, would establish that the sham baseline is sound across the full range of stimuli to which this protocol has been applied.

## 5. Conclusions

Anodal tDCS delivered through the Fp3–Fp2 montage reduces the FIE, and it does so by impairing recognition of upright faces specifically. The beliefs that accompany taking part in this stimulation study, as distinct from expectations that are measured or deliberately induced, had no detectable effect on performance. The reduction in the FIE is therefore unlikely to be attributable to those beliefs, and within the paradigm and procedure employed here the sham control on which a decade of work with this protocol has depended can be treated as the baseline it was assumed to be.

## Figures and Tables

**Table 1:** Means and standard deviations of *d*′ by group and face orientation.

		Upright		Inverted		Inversion Effect	
Group	*n*	*M*	*SD*	*M*	*SD*	*M*	*SD*
Anodal	32	0.44	0.23	0.21	0.25	0.23	0.34
Sham	32	0.75	0.23	0.21	0.23	0.55	0.31
No-tDCS	32	0.77	0.28	0.22	0.21	0.56	0.35

**Table 2:** Between-group comparisons and Bayes factors.

Comparison	*M* Difference	*t*(62)	p	BF	BF_10_
Inversion effect					
Anodal vs. Sham	−0.32	−3.92	<0.001	659	111
Anodal vs. No-tDCS	−0.33	−3.79	<0.001	401	75.6
Sham vs. No-tDCS	−0.01	−0.10	0.922	0.27	0.26
Upright d′					
Anodal vs. Sham	−0.31	−5.40	<0.001	>1000	>1000
Anodal vs. No-tDCS	−0.33	−5.17	<0.001	>1000	>1000
Sham vs. No-tDCS	−0.02	−0.29	0.775	0.25	0.27
Inverted d′					
Anodal vs. Sham	0.01	0.13	0.900	0.25	0.26
Anodal vs. No-tDCS	−0.00	−0.05	0.961	0.22	0.26
Sham vs. No-tDCS	−0.01	−0.19	0.852	0.20	0.26
Overall d′					
Anodal vs. Sham	−0.15	−3.58	<0.001	167	43.5
Anodal vs. No-tDCS	−0.17	−3.89	<0.001	515	102
Sham vs. No-tDCS	−0.01	−0.33	0.742	0.18	0.27

## Data Availability

The data underlying this article will be shared on reasonable request to the corresponding author and are available in an open repository (https://osf.io/qyz2d/overview, accessed on 26 August 2026).
